# Effects of Tobacco Use on Oral Cancer Screening Algorithm Performance

**DOI:** 10.3390/cancers18010176

**Published:** 2026-01-05

**Authors:** Elyse Kanagandram, Aksel Alp, Thair Takesh, Cherie Wink, Susan Yang, Amber Davis, Michelle Hurlbutt, Jerica Block, Petra Wilder-Smith

**Affiliations:** 1 Beckman Laser Institute, University of California Irvine School of Medicine, Irvine, CA 92612, USA; 2 School of Dental Hygiene, San Joaquin Valley College, Ontario, CA 91764, USA; 3 School of Dental Hygiene, Stanbridge University, Riverside, CA 92507, USA; 4 School of Dental Hygiene, West Coast University, Anaheim, CA 92802, USA

**Keywords:** oral cancer, screening, algorithm, oral lesion, tobacco, risk factors

## Abstract

**Background/Objectives:** Effective screening for oral cancer (OC) remains challenging. Inaccuracies contribute to delayed diagnosis and poor outcomes. Tobacco-related changes in oral mucosa may compromise the accuracy of oral screening approaches, and, in emerging “smart” screening modalities, they may overshadow the influence of other predictive variables. The objective of this study was to evaluate the screening accuracy of an imaging- and risk factor-based OC screening platform in individuals practicing different types of tobacco usage. **Methods:** 318 subjects who had previously screened positive for increased OC risk were recruited and sorted into “tobacco smoker”, “tobacco vaper”, “tobacco chewer”, “hookah user”, “multiple tobacco usage”, or “tobacco non-user” groups. Next, demographic information, risk factors, outcome of clinical examination, as well as AFI and pWLI were recorded using a prototype OC screening platform. The OC risk assessment outcome from the OC screening platform was compared to that from an oral medicine specialist. **Results:** The screening platform demonstrated high sensitivity in tobacco chewers and hookah users, and it also exceeded 90% in smokers, vapers, and multi-product users. In tobacco non-users, 80% screening sensitivity was recorded. Screening specificity was considerably better in tobacco non-users than in the tobacco-user groups, and low in tobacco chewers (33.3%), vapers (55.6%), and smokers (62.5%). Across all groups, agreement between the screening platform outcome and specialist evaluation exceeded 80%. Significant differences in probe accuracy were noted between tobacco non-users and users (*p* < 0.05), except for tobacco vapers. **Conclusions:** These findings highlight the need to consider the effects of type of tobacco use on the OC screening approach, and to integrate these variables into imaging-and risk-factor-based algorithms for OC screening.

## Simple Summary

The aim of this clinical study was to compare the accuracy of an automated oral cancer screening platform in individuals with different modes of tobacco use. The reason for this study is that the tobacco-related changes in the soft tissues of the mouth vary depending on the type of tobacco that is being used. The findings from this research indicate that screening accuracy is greatly affected by the type of tobacco use practiced. Thus, in the future, this variable needs to be included in oral cancer screening approaches to ensure more accurate detection of oral cancer risk, resulting in earlier referral and better treatment outcomes.

## 1. Introduction

Oral cancer (OC) remains a significant global health issue, with an estimated 377,713 new cases and 177,757 deaths reported worldwide every year [1]. In the United States alone, approximately 54,000 new cases and 11,000 deaths are attributed to OC annually [2]. OC exhibits notably higher mortality and morbidity than many major cancers, surpassing other common cancers such as cervical, laryngeal, Hodgkin’s lymphoma, and many others [3]. The five-year survival rate for OC in the U.S. is approximately 62%, with the patient’s prognosis heavily influenced by the disease stage at diagnosis [3]. The majority of cases are identified at advanced stages, when metastasis has occurred, complicating treatment and leading to poor outcomes with high morbidity and mortality [3,4,5,6,7,8].

The reason for the predominantly late diagnosis of most oral squamous cell carcinomas (OSCCs) is attributed to the subtle and asymptomatic nature of oral potentially malignant (OPML) and early-stage lesions, which often mimic benign conditions. These similarities in presentation hinder early detection and are considered a primary cause of referral delay [4,5,9]. Barriers to effective risk screening are particularly pronounced in tobacco users, in whom other tobacco use-related mucosal changes often mask clinical manifestations of OPMLs or OCs.

OPMLs carry a heightened risk of malignant transformation in tobacco users compared to non-users [10,11,12,13,14]. Consequently, tobacco use is recognized as one of the most significant etiological risk factors for OC, with 75–90% of individuals diagnosed with OC having a history of tobacco consumption [3,15,16,17,18,19,20]. Studies indicate that tobacco smokers are five times more likely to develop OC than nonsmokers, with smokeless tobacco also increasing OC risk, particularly at the sites of tobacco placement in the mouth [5,21,22,23,24]. Different forms of tobacco use—including smoking, chewing, vaping, and hookah smoking—are associated with varying usage patterns and health risks [25,26,27,28,29,30,31,32,33,34,35,36,37]. Moreover, mucosal manifestations vary considerably between different types of tobacco use, yet existing screening tools are not tailored to these differences [25,26,27,28,29,30,31,32,33,34,35,36,37]. Tobacco smoking remains the most common form of consumption, followed by vaping, chewing, and hookah smoking [38]. The primary dysplastic oral lesions linked to tobacco use include leukoplakia, erythroplakia, and oral submucous fibrosis [27,28,29,30,31,32,33,34,35,39]. OSCC accounts for over 90% of oral cancer cases and is often preceded by OPMLs, which encompass a variety of mucosal and submucosal abnormalities, with a malignant transformation rate up to 10% per year [5,39,40,41,42,43].

Low socioeconomic status (SES) is strongly associated with increased tobacco use, placing this demographic at heightened risk for OC and OPMLs. Indeed, individuals with a low SES carry the highest risk of OC in the United States [44,45,46,47,48,49,50,51,52,53,54,55,56,57,58,59,60]. Minority groups and those living in remote areas also face elevated OC risks due to higher tobacco use, low health literacy, and limited access to healthcare [44,58,61,62,63]. Minority disparities in five-year survival rates are evident, with survival rates over five years measuring 66% and 71%, respectively, in white men and women, compared to 56% and 64% in black men and women [64]. On a global scale, low- and middle-income countries (LMICs), particularly in Southeast Asia and India, face significant OC burdens due to high areca and betel-nut-based tobacco consumption, which often begins at a young age [61,62,65,66,67,68,69]. India alone accounts for approximately one-quarter of global OC cases, with a five-year survival rate of only 50%, compared to 68% in developed countries [3,68,70]. Healthcare facilities in LMICs are predominantly urban-centered, leaving low- and middle-income populations, who typically reside in rural areas, at a significant disadvantage due to limited access to screening, diagnosis, and care [62,65].

Clinical oral examination (COE), the current standard for OC screening, has limited accuracy due to the similar presentation of many common non-cancerous conditions [71,72,73]. Its findings are often subjective and heavily reliant on the clinician’s experience and expertise [39,73,74,75,76,77,78,79,80,81,82,83,84,85,86,87]. Various adjunctive screening tools exist; however, their effectiveness remains ambiguous [11,88,89,90,91,92,93], and they are not currently recommended [39]. A mobile health (mHealth) program has shown promise in assisting community healthcare workers (CHWs) in India with screening effectiveness and implementation in remote populations [94]. This technology-driven platform incorporates a questionnaire, image-capturing technology, and rapid data transmission to specialists, enabling frontline, non-specialist clinicians to screen individuals in low-resource and remote settings [94].

Recent advances in artificial intelligence (AI) present promising opportunities to enhance OC screening accuracy, particularly through combined autofluorescence imaging (AFI) and polarized white light imaging (pWLI) [92,95,96,97,98,99]. When integrated with AI, these optical imaging modalities create “smart”, cost-effective screening platforms that can easily be used by non-specialists to enhance early detection and, ultimately, improve oral cancer outcomes [97,98,99,100,101,102,103].

Our overall objective is to improve OC outcomes by developing an effective and accurate smartphone-based screening platform for non-specialist use in low-resource communities. This platform aims to assess OC risk levels by integrating multimodal intraoral imaging, clinical signs and symptoms, and relevant risk factors. The specific objective of this study was to evaluate the impact of various forms of tobacco use on the accuracy of a prototype imaging- and risk factor-based OC screening platform.

## 2. Materials and Methods

This study was conducted in accordance with the Declaration of Helsinki and approved by the Institutional Review Board of the University of California Irvine (protocol number 2002-2805) for studies involving humans. Written informed consent was obtained from all subjects involved in the study, all of whom completed the study in full compliance with the approved protocol.

### 2.1. Subjects

A total of 318 participants who had screened positive for increased OC risk at Concorde College of Dental Hygiene in Garden Grove, CA, West Coast University Dental Hygiene Clinic in Anaheim, CA, and the University of California, Irvine’s Clinics were recruited and classified into ‘tobacco smoker’, ‘tobacco vaper’, ‘tobacco chewer’, ‘hookah user’, ‘multiple tobacco usage ’, or ‘tobacco non-user’ groups. Subjects self-categorized their tobacco user status. Participants in each tobacco use category had engaged in their reported tobacco use habits for at least 1 year prior to their participation in this study. Subject demographics are shown in Table 1.

### 2.2. Oral Cancer Screening Platform

**Figure 1:**
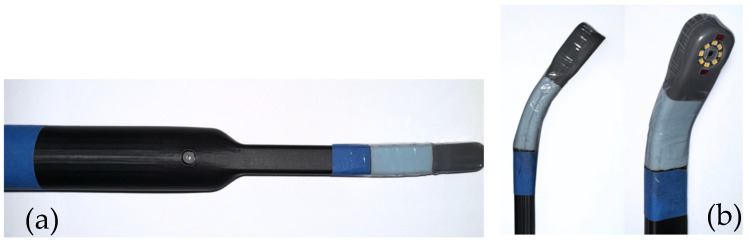
Handheld prototype intra-oral scanner system designed and built by our team: (**a**) scanner device with extended reach to improve intraoral access, (**b**) flexible tip permitting imaging access to all intra-oral sites, including base of tongue and tonsillar region.

A prototype oral health screening platform whose technology and algorithm were previously developed by our group was used in this study (Figure 1). It was connected wirelessly to a Dell laptop computer (Dell, Round Rock, TX, USA) on which the prototype software and user interface had been installed. The platform was used by the same experienced dental hygienist throughout this study to collect and record all the data and images that were subsequently analyzed by the screening algorithm.

Demographic information, OC risk factors, as well as polarized white light (pWLI) and autofluorescence (AFI) images from the prototype intra-oral scanner were recorded using the platform’s App. These data were then automatically uploaded to a secure, cloud-based, HIPAA-compliant site, where a prototype algorithm processed them and registered an OC risk categorization for each intra-oral site. The algorithmic triage output had 3 categories: ‘no increased risk’, ‘moderate risk’, or ‘high’ risk, and these were displayed on the linked laptop computer.

### 2.3. Protocol

**Figure 2:**
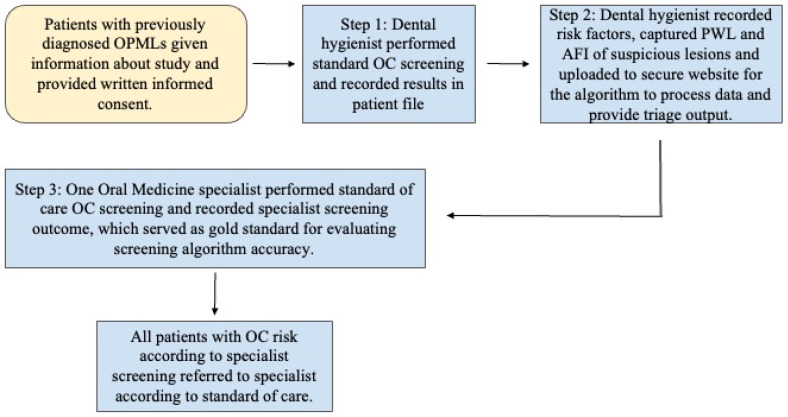
Flow diagram of study protocol.

Patients who had previously screened positive for OPMLs were provided full details about this study, provided with the opportunity to ask questions of the study team, and then they were invited to participate in the study. Individuals were told that non-participation would not affect their treatment in any way. Those who opted into the study provided written informed consent according to the approved IRB protocol. Data collection included 3 separate steps. First, the dental hygienist completed a standard oral cancer screening process according to her daily clinical practice and as taught at dental hygiene schools. This includes a clinical exam and risk factor assessment. These findings were recorded in the patient’s file. The same experienced, pre-standardized hygienist completed all examinations and all data collection throughout the study. She had previously obtained 94% accuracy for clinical recognition of lesions using a biopsy-benchmarked database of 200 images of oral lesions. The second step of the protocol encompassed data collection using the screening platform. First, the hygienist completed a de novo standard oral cancer risk habit assessment and recorded the results directly on the platform App. Next, she acquired pWLI and AFI images of any potentially suspicious lesions, and these, too, were recorded on the platform App. The automated HIPAA-compliant system then uploaded all the recorded data to our proprietary, secure website, where a previously developed algorithm processed the data and provided triage output. In the third step of the data collection process, one oral medicine specialist who was not aware of the previous screening outcomes from the hygienist and the automated algorithm performed a full standard of care OC screening. The specialist subsequently recorded this screening outcome in conventional patient records as either “no increased risk”, “moderate risk”, or “high risk” for each study participant. The same specialist performed all screening in all participants according to the standard of care, combining clinical examination with risk factors and patient history. This specialist screening outcome was used as the gold standard to subsequently evaluate the accuracy of the screening algorithm in each patient and for each lesion. Finally, the standard of care specialist screening outcome was communicated to all study participants, and they underwent referral in accordance with that outcome and in accordance with the standard of care (Figure 2).

### 2.4. Statistical Analysis

Data were analyzed using standard SPSS 19 software (IBM^®^, Armonk, NY, USA). Sensitivity, specificity, specialist agreement, false positive rate, false negative rate, and positive and negative predictive values were estimated from the observed results. Standard errors (SE) and 95% confidence intervals were calculated for all the rates. A level of *p* < 0.05 was used to achieve statistical significance.

## 3. Results

1099 AFI and pWLI image pairs were analyzed by the prototype algorithm in combination with the matching risk factor data. The algorithm processed these datasets and then indicated the screening outcome on the laptop screen: either no increased risk, moderate risk, or high risk.

### Screening Accuracy vs. Specialist (Gold Standard)

Table 2 summarizes a comparison of the screening algorithm performance vs. the screening outcome from the oral medicine specialist in tobacco non-users and tobacco smokers. Briefly, the algorithm achieved a higher sensitivity but a lower specificity and a slightly higher agreement with specialist screening outcome in tobacco smokers vs. non-smokers. Its output performance achieved 90% sensitivity, 62.5% specificity, and 84.2% agreement with the specialist screening outcome in tobacco smokers, and 80% sensitivity, 100% specificity, and 82.4% agreement with the specialist screening outcome in tobacco non-users. The remaining performance measures are summarized in Table 2.

Table 3 provides a comparison of the screening performance of the algorithm vs. the oral medicine specialist in tobacco vapers. The algorithm achieved 93.3% sensitivity, 55.6% specificity, and 84.6% agreement with the specialist screening outcome in tobacco vapers. A more detailed breakdown of the results is shown in Table 3.

Table 4 details screening algorithm performance in tobacco chewers. The algorithm achieved a very high level of sensitivity (100%) and a good level of agreement with the specialist screening outcome (81.8%) but a remarkably low level of specificity in its triage guidance (33.3%). A comprehensive summary of the statistical analysis is presented in Table 4.

Table 5 provides an overview of the screening algorithm performance in tobacco hookah smokers. While the algorithm achieved 100% sensitivity and 86.7% agreement with the specialist screening outcome in this population, its specificity was only moderate (71.4%). A more extensive analysis of the data is shown in Table 5.

Table 6 presents an overview of the performance of the screening algorithm in multi-tobacco users. In this group, too, the algorithm achieved a good sensitivity of 95.2% and reasonable agreement (81.5%) agreement with the specialist screening outcome. However, the specificity of the algorithm was poor (33.3% specificity). A full overview of the performance measures is presented in Table 6.

A summary of the overall performance of the screening algorithm in all tobacco users combined is summarized in Table 7. Briefly, the algorithm achieved 94.3% sensitivity, 52.8% specificity, and 83.7% agreement with the specialist screening outcome.

Table 8 provides a summary of the screening platform performance in all the tobacco use groups included in this study. Agreement between the specialist screening outcome and the probe screening output varied considerably between the different risk groups.

The algorithm achieved moderate levels of agreement with the gold standard in all tobacco use categories, with a high level of risk except for tobacco non-users, where agreement with specialist screening outcome was only 66.7%. In individuals with a gold standard screening outcome of “moderately increased risk”, agreement between the specialist and the algorithm ranged from 66.7 to 84.0%, with a mean value of 80.2%. Algorithm performance was poorest in tobacco chewers. Finally, in individuals with a gold standard screening outcome of “no increased risk”, agreement between the algorithmic and the specialist screening outcome ranged from 66.7 to 100%, with the algorithm performing best in tobacco non-users and vapers, while it performed most poorly in tobacco chewers and multi-users.

Screening accuracy by the algorithm differed significantly between tobacco non-users and each tobacco use group (*p* < 0.05), except for the vaping group, where screening accuracy did not differ significantly from that observed in the tobacco non-user group.

## 4. Discussion

The goal of this study was to deepen our understanding of the effect of different tobacco-use types on the performance of an AI-generated imaging- and risk-factor-based screening algorithm for OC risk. Our previous research had compared the screening accuracy of a “smart” OC screening platform in tobacco non-users vs. tobacco users [99]. However, it did not examine the influence of different types of tobacco consumption, such as vaping, chewing, and hookah smoking on automated screening outcomes. Because daily usage habits and clinical oral manifestations are changing [104], and can differ considerably between the various types of tobacco consumption, it seemed reasonable to assume that these differences might affect the variables that are collected by the screening platform, the weighting of these variables within the algorithm architecture, and hence also the accuracy of the screening algorithm output. Multiple studies have indeed confirmed that the accuracy of oral cancer screening by conventional methods is affected by tobacco use [105]. Another study reported that the chronic inflammatory, white, and/or red lesions as well as keratotic changes that can be related to tobacco use can closely resemble potentially malignant disease and can therefore increase false positives [106].

Findings from the current study revealed that the type of tobacco use does indeed influence greatly the screening accuracy of the “smart” OC screening platform. While agreement with the specialist screening outcome remained consistently above 80% across all groups, and the automated system tested in this study manifested overall high screening sensitivity, specificity was low in chewers, vapers, and smokers relative to the much higher specificity observed in tobacco non-users. These outcomes are supported by reports from other studies that optically based screening modalities are especially susceptible to the confounding effects of tobacco-related mucosal changes [105]. The risk of poor screening accuracy is further compounded when such devices use only one wavelength of autofluorescence to assess OC risk, without incorporating risk factors and habits and/or other optical parameters or multiple wavelengths of light [106].

To the best of our knowledge, there exists little information to date regarding the specific effects of different types of tobacco products on screening accuracy, nor has the role of variables such as frequency and duration of tobacco usage been elucidated. While our previous studies have investigated extensively the design and technology as well as the merits and disadvantages of various algorithmic approaches [107,108,109,110,111,112], the results of this study suggest that further refinement of the algorithm—particularly in calibrating the “weight” or “impact” of each tobacco-related variable within the algorithm architecture—should be considered. Moreover, expanded training data—to take into account the frequency of tobacco use, and to capture a broader spectrum of mucosal presentations that may co-exist with tobacco-related lesions—should be included in the next stages of algorithm refinement.

In this study, on-site specialist screening served as the gold standard. This approach was adopted in order to provide ready access to a wide range of tobacco users in whom biopsy would not be considered appropriate according to the standard of care. However, given the potentially negative effect of tobacco use on conventional screening outcomes, additional studies that use biopsy results as the gold standard are needed to provide a more accurate and differentiated determination of the screening platform’s performance.

Overall, the findings of this study highlight the multifaceted impact of tobacco use on oral mucosal status, with each form of consumption presenting distinct screening challenges. The results underscore the importance of further refining the AI-based screening algorithm to ensure that it provides the most accurate screening outcomes possible as an important step in the pathway to improving early oral cancer detection and treatment outcomes.

## 5. Conclusions

This study highlights the need to consider the type of tobacco use during OC screening, as this variable considerably affects screening accuracy when an imaging and risk factor-based OC screening algorithm is used.

## Figures and Tables

**Table 1:** Subject demographics.

		Tobacco Smoker	Tobacco Vaper	Tobacco Chewer	Hookah	Multiple	Tobacco Non-User
Mean age	52	63	28	41	48	54	51
Age range	21–82	21–82	21–36	23–66	31–62	21–66	21–79
African American	21	8	4	4	0	1	4
American Indian/Alaska Native	0	0	0	0	0	0	0
Asian	112	33	41	5	1	16	16
Multiple Categories	31	6	9	4	6	1	5
Native Hawaiian or Pacific Islander	4	1	2	0			1
White Hispanic	102	41	32	6	0	10	13
White non-Hispanic	48	9	11	6	10	5	7
Female	117	37	33	0	3	11	33
Male	201	55	57	21	8	21	39
Non-binary	0						
TOTAL	318	98	99	25	17	33	46

**Table 2:** Accuracy of screening algorithm in non-tobacco users and tobacco smokers.

		VALUE	SE	LOWER CI	UPPER CI
No Tobacco	Sensitivity	0.800	0.073	0.657	0.943
	Specificity	1.000	0.000	1.000	1.000
	FNR (False Negative Rate)	0.200	0.073	0.057	0.343
	FPR (False Positive Rate)	0.000	0.000	0.000	0.000
	Agreement with specialist	0.824	0.065	0.695	0.952
	PPV (Positive Predictive Value)	1.000	0.000	1.000	1.000
	NPV (Negative Predictive Value)	0.400	0.155	0.096	0.704
Tobacco Smoker	Sensitivity	0.900	0.055	0.793	1.007
	Specificity	0.625	0.171	0.290	0.960
	FNR (False Negative Rate)	0.100	0.055	−0.007	0.207
	FPR (False Positive Rate)	0.375	0.171	0.040	0.710
	Agreement with specialist	0.842	0.059	0.726	0.958
	PPV (Positive Predictive Value)	0.900	0.055	0.793	1.007
	NPV (Negative Predictive Value)	0.625	0.171	0.290	0.960

**Table 3:** Accuracy of screening algorithm in tobacco vapers.

		VALUE	SE	LOWER CI	UPPER CI
Tobacco Vaper	Sensitivity	0.933	0.046	0.844	1.023
	Specificity	0.556	0.166	0.231	0.880
	FNR (False Negative Rate)	0.067	0.046	−0.023	0.156
	FPR (False Positive Rate)	0.444	0.166	0.120	0.769
	Agreement with specialist	0.846	0.058	0.733	0.959
	PPV (Positive Predictive Value)	0.875	0.058	0.760	0.990
	NPV (Negative Predictive Value)	0.714	0.171	0.380	1.049

**Table 4:** Accuracy of screening algorithm in tobacco chewers.

		VALUE	SE	LOWER CI	UPPER CI
Tobacco Chewer	Sensitivity	1.000	0.000	1.000	1.000
	Specificity	0.333	0.192	−0.044	0.711
	FNR (False Negative Rate)	0.000	0.000	0.000	0.000
	FPR (False Positive Rate)	0.667	0.192	0.289	1.044
	Agreement with specialist	0.818	0.082	0.657	0.979
	PPV (Positive Predictive Value)	0.800	0.089	0.625	0.975
	NPV (Negative Predictive Value)	1.000	0.000	1.000	1.000

**Table 5:** Accuracy of screening algorithm in tobacco hookah smoker.

		VALUE	SE	LOWER CI	UPPER CI
Tobacco Hookah	Sensitivity	1.000	0.000	1.000	1.000
	Specificity	0.714	0.171	0.380	1.049
	FNR (False Negative Rate)	0.000	0.000	0.000	0.000
	FPR (False Positive Rate)	0.286	0.171	−0.049	0.620
	Agreement with specialist	0.867	0.088	0.695	1.039
	PPV (Positive Predictive Value)	0.800	0.126	0.552	1.048
	NPV (Negative Predictive Value)	1.000	0.000	1.000	1.000

**Table 6:** Accuracy of screening algorithm in tobacco multi-users.

		VALUE	SE	LOWER CI	UPPER CI
Tobacco Multi-users	Sensitivity	0.952	0.046	0.861	−0.044
	Specificity	0.333	0.192	1.043	0.711
	FNR (False Negative Rate)	0.048	0.046	−0.043	0.139
	FPR (False Positive Rate)	0.667	0.192	0.289	1.044
	Agreement with specialist	0.815	0.075	0.668	0.961
	PPV (Positive Predictive Value)	0.833	0.076	0.684	0.982
	NPV (Negative Predictive Value)	0.667	0.272	0.133	1.200

**Table 7:** Accuracy of screening algorithm in all tobacco users.

		VALUE	SE	LOWER CI	UPPER CI
Tobacco-All	Sensitivity	0.943	0.023	0.898	0.987
	Specificity	0.528	0.083	0.365	0.691
	FNR (False Negative Rate)	0.057	0.023	0.013	0.309
	FPR (False Positive Rate)	0.472	0.083	0.102	0.635
	Agreement with specialist	0.837	0.031	0.776	0.898
	PPV (Positive Predictive Value)	0.853	0.033	0.789	0.593
	NPV (Negative Predictive Value)	0.760	0.085	0.918	0.927

**Table 8:** Accuracy of screening algorithm in all groups.

	No Increased Risk	Moderate Increased Risk	High Risk	*p*-Value
No Tobacco	1.000	0.833	0.667	
Smoker	0.833	0.840	0.857	0.0478
Vaper	1.000	0.793	1.000	0.068
Chewer	0.667	0.667	1.000	0.0003
Hookah user	0.833	0.800	1.000	0.007
Multi user	0.667	0.769	0.909	0.0016
All users	0.826	0.802	0.946	0.0145

## Data Availability

Data is unavailable due to privacy or ethical restrictions.
